# Cytosolic 5′-nucleotidase 1A autoantibody profile and clinical characteristics in inclusion body myositis

**DOI:** 10.1136/annrheumdis-2016-210282

**Published:** 2017-01-25

**Authors:** J B Lilleker, A Rietveld, S R Pye, K Mariampillai, O Benveniste, M T J Peeters, J A L Miller, M G Hanna, P M Machado, M J Parton, K R Gheorghe, U A Badrising, I E Lundberg, S Sacconi, M K Herbert, N J McHugh, B R F Lecky, C Brierley, D Hilton-Jones, J A Lamb, M E Roberts, R G Cooper, C G J Saris, G J M Pruijn, H Chinoy, B G M van Engelen

**Affiliations:** 1 Centre for Musculoskeletal Research, Division of Musculoskeletal and Dermatological Sciences, School of Biological Sciences, Faculty of Biology, Medicine and Health, Manchester Academic Health Science Centre, The University of Manchester, Manchester, UK; 2 Greater Manchester Neurosciences Centre, Salford Royal NHS Foundation Trust, Stott Lane, Salford, UK; 3 Department of Neurology, Center for Neuroscience Donders Institute for Brain, Cognition and Behaviour, Radboud University Medical Center, Nijmegen, The Netherlands; 4 Department of Internal Medicine and Clinical Immunology, La Pitié-Salpêtrière Hospital, AP-HP, INSERM U974, UPMC, Paris, France; 5 Department of Neurology, Royal Victoria Hospitals, The Newcastle upon Tyne Hospitals NHS Foundation Trust, Newcastle, UK; 6 MRC Centre for Neuromuscular Diseases, Institute of Neurology, University College London, London, UK; 7 Centre for Rheumatology Research, University College London, London, UK; 8 Unit of Rheumatology, Department of Medicine, Karolinska University Hospital, Solna, Karolinska Institutet, Stockholm, Sweden; 9 Department of Neurology, Leiden University Medical Center, Leiden, The Netherlands; 10 Peripheral Nervous System, Muscle and ALS Department, Université Côté Azure (UCA), Nice University Hospital, Nice, France; 11 Department of Biomolecular Chemistry, Radboud Institute for Molecular Life Sciences and Institute for Molecules and Materials, Radboud University, Nijmegen, The Netherlands; 12 Royal National Hospital for Rheumatic Diseases and Department of Pharmacy and Pharmacology, University of Bath, Bath, UK; 13 The Walton Centre NHS Foundation Trust, Fazakerley, Liverpool, UK; 14 Department of Neurology, Cambridge University Hospitals NHS Foundation Trust, Cambridge, UK; 15 Nuffield Department of Clinical Neurosciences, Oxford University Hospitals, Oxford, UK; 16 Centre for Integrated Genomic Medical Research, University of Manchester, Manchester, UK; 17 MRC-ARUK Institute for Ageing and Chronic Disease, University of Liverpool, Liverpool, UK; 18 Rheumatology Department, Salford Royal NHS Foundation Trust, Salford, UK; 19 NIHR Manchester Musculoskeletal Biomedical Research Unit, Central Manchester University Hospitals NHS Foundation Trust, Manchester Academic Health Science Centre, Manchester, UK

**Keywords:** Autoantibodies, Dermatomyositis, Polymyositis

## Abstract

**Objectives:**

Autoantibodies directed against cytosolic 5′-nucleotidase 1A have been identified in many patients with inclusion body myositis. This retrospective study investigated the association between anticytosolic 5′-nucleotidase 1A antibody status and clinical, serological and histopathological features to explore the utility of this antibody to identify inclusion body myositis subgroups and to predict prognosis.

**Materials and methods:**

Data from various European inclusion body myositis registries were pooled. Anticytosolic 5′-nucleotidase 1A status was determined by an established ELISA technique. Cases were stratified according to antibody status and comparisons made. Survival and mobility aid requirement analyses were performed using Kaplan-Meier curves and Cox proportional hazards regression.

**Results:**

Data from 311 patients were available for analysis; 102 (33%) had anticytosolic 5′-nucleotidase 1A antibodies. Antibody-positive patients had a higher adjusted mortality risk (HR 1.89, 95% CI 1.11 to 3.21, p=0.019), lower frequency of proximal upper limb weakness at disease onset (8% vs 23%, adjusted OR 0.29, 95% CI 0.12 to 0.68, p=0.005) and an increased prevalence of excess of cytochrome oxidase deficient fibres on muscle biopsy analysis (87% vs 72%, adjusted OR 2.80, 95% CI 1.17 to 6.66, p=0.020), compared with antibody-negative patients.

**Interpretation:**

Differences were observed in clinical and histopathological features between anticytosolic 5′-nucleotidase 1A antibody positive and negative patients with inclusion body myositis, and antibody-positive patients had a higher adjusted mortality risk. Stratification of inclusion body myositis by anticytosolic 5′-nucleotidase 1A antibody status may be useful, potentially highlighting a distinct inclusion body myositis subtype with a more severe phenotype.

## Introduction

Inclusion body myositis (IBM) is an acquired muscle disease that most commonly affects males aged over 45 years. Along with polymyositis (PM) and dermatomyositis (DM), IBM is usually classified as one of the idiopathic inflammatory myopathies. However, IBM differs in comparison with PM and DM, as sustained responses to immunosuppression are not seen, and histologically it is associated with significant degenerative features.^1–3^ Clinically, IBM is characterised by asymmetric weakness, notably of finger flexors and knee extensors. Weakness in other muscle groups occurs frequently, including bulbar, facial and axial muscles.^4^ ^5^ The slowly progressive course leads to cumulative disability, although overall life expectancy is unaffected.^6–8^

The diagnosis of IBM relies upon a combination of clinical and laboratory findings as defined in various diagnostic criteria (eg, Medical Research Council (MRC), Griggs *et al* and the European Neuromuscular Centre (ENMC) criteria).^9–11^ However, certain histopathological findings may only become detectable as the disease progresses, and therefore patients with early disease may not fulfil definite diagnostic criteria and can be excluded from clinical trials.^12^ The average delay between disease onset and diagnosis is around 5 years, and IBM is frequently misdiagnosed initially as PM, resulting in the unnecessary use of potentially harmful treatments, such as high-dose glucocorticoids.^8^ ^13–15^

In IBM, autoantibodies directed against cytosolic 5′-nucleotidase 1A (cN-1A) have recently been identified. It is suggested that these may support the diagnostic process, as well as potentially providing clues as to disease pathogenesis.^16^ ^17^ However, uncertainties regarding the usefulness of anti-cN-1A autoantibody testing in clinical practice remain. This is particularly true with regard to patient stratification and prognosis, where the few studies that have compared clinical and histopathological features of antibody-positive versus antibody-negative patients with IBM have produced conflicting results in some cases.^18^ ^19^ In order to explore further the usefulness of anti-cN-1A antibody testing to facilitate IBM subgroup classification, we conducted a retrospective Europe-wide study correlating clinical, serological and histopathological features in a large cohort of patients with IBM stratified by anti-cN-1A antibody status.

## Patients and methods

### Study cohort

Pooled IBM case data from four European countries were used. Researchers based in Nijmegen, The Netherlands, coordinated data collection from The Netherlands, France and Sweden. Data collection in the UK was coordinated by researchers based in Manchester, UK.

### Study inclusion criteria

Included cases met either the MRC (‘pathologically defined’, ‘clinically defined’ or ‘possible’), Griggs *et al* (‘definite’ or ‘possible’) or ENMC (‘clinicopathologically defined’, ‘clinically defined’ or ‘probable’) diagnostic criteria for IBM and had sera available for anti-cN-1A antibody testing.^9^ ^11^

### Data collection methodology

Swedish, French and Dutch (‘non-UK’) patients were identified from clinical databases. Researchers blinded to anti-cN-1A antibody status (AR, MTJP, KRG, KM) reviewed the medical records and retrospectively completed a standardised data collection pro forma. UK patients were identified from six centres contributing to the UKMYONET research study, coordinated by The University of Manchester. As part of this study, data are captured using a standardised pro forma at the time of study recruitment (ie, before serological test results are available).^20^ ^21^ Those recruiting patients are asked to record clinical features present at disease onset and features present at the time of recruitment. Some additional fields (to match data from the non-UK cohort) and missing data were collected retrospectively. Copies of pro forma used are contained in online supplementary appendix 1. The datasets were merged and cleaned by a researcher blinded to anti-cN-1A status (JBL).

10.1136/annrheumdis-2016-210282.supp1 supplementary appendix

### Clinical data

Data collected included demographic, clinical (eg, distribution of weakness, presence of dysphagia, comorbidities), laboratory findings (creatine kinase (CK) levels, muscle biopsy features, serological testing), comorbidity, mobility aid usage and mortality. In most cases, data were available regarding features present at disease onset and at the time of last patient review (or recruitment to the UKMYONET study in the case of the UK cohort). In all cases, ‘disease onset’ refers to the initial date that symptoms of IBM were noted, as reported by the patient. ‘Disease duration’ is defined as the period between disease onset and the date of anti-cN-1A antibody testing. Regarding mortality, in the non-UK cohort, the primary cause of death was categorised by review of the patient's medical records as either ‘respiratory’, ‘cardiac’, ‘cerebrovascular’, ‘malignancy’ or ‘other’. In the UK cohort, additional mortality and comorbidity statistics were obtained from the UK Health and Social Care Information Centre, including coded data regarding the cause of death where applicable. The cause of death in these cases was assessed and assigned to the same categories as the non-UK cohort.

### Histopathology

For all cases, the histopathology biopsy report performed at initial diagnostic interrogation was reviewed, and the presence of certain specific features determined from the report text. The reporting histopathologists were blinded to the anti-cN-1A antibody status of each patient at the time of reporting. Cytochrome oxidase (COX) deficient fibres in the biopsy sample were recorded as ‘excessive’ if the reporting histopathologist indicated that numbers were adjudged higher than expected, according to the patient’s age. In some cases, the date that the biopsy was performed was not available. In such instances, this was assumed to be the same as the date of diagnosis.

### cN-1A analysis

All sera were analysed at the Department of Biomolecular Chemistry in Nijmegen by ELISA, with the three synthetic peptides containing cN-1A autoepitopes previously identified by overlapping peptide microarray analyses.^16^ Signals were quantified by determining optical densities at 450 nm (OD450) using methods previously described and defined as seropositive if the OD450 value was greater than or equal to the established cut-off value for the corresponding peptide.^22^

### Other serological testing

Data regarding the presence of myositis-specific antibodies (MSAs) and myositis-associated antibodies (MAAs) were collected where available. For the non-UK patients, data were obtained from results available in the medical records, and methodology of testing was unique to each centre. MSAs and MAAs in the whole UK cohort were screened by immunoprecipitation at the University of Bath (Bath, UK) using previously described standardised methodology.^23^ ‘Weak positive’ results were assumed to be negative for the purpose of this study.

### Statistical analysis

The per-subject sum of all recorded comorbidities (of autoimmune disease, cardiovascular disease (including hypertension) and malignancy) was calculated. Current or previous smoking was also treated as a comorbidity for the purposes of this analysis. According to the number of these factors present, each patient was then assigned a comorbidity score of 0, 1 or 2 or more for use in regression. Differences in demographic features, comorbidities, clinical features, autoantibody status and muscle biopsy features between anti-cN-1A antibody positive and negative patients were assessed using logistic regression. In order to test the effect of potential confounders, adjusted (multivariable) logistic regression models were produced when unadjusted analysis had suggested a significant difference (defined as p<0.05).

The impact of anti-cN-1A antibody status on survival and mobility aid requirement was assessed using Kaplan-Meier curves, log-rank testing and Cox proportional hazards regression modelling. In both cases, the start of the surveillance period was the date of disease onset. For the mobility aid analysis, subjects exited the model at the time of mobility aid requirement or at the time they were last known to have not required one. For the survival analysis, subjects exited the model at the time of death or at the time they were last known to have been alive. Each Cox regression model included adjustment for age of disease onset, gender and comorbidities. Other variables were added to the models if there was an a priori assumption that a relationship between anti-cN-1A antibody status and the outcome variable was likely to exist. For example, a higher incidence of anti-cN-1A antibodies in those with Sjögren's syndrome is reported, a more prominent bulbar involvement in anti-cN-1A positive patients with IBM has been described and a correlation between COX deficiency and more advanced age at biopsy could exist.^18^ ^22^ ^24^ Therefore, models with additional adjustment for such variables were created.

The analysis plan specifically omitted correction for multiple testing due to the highly conservative nature of such methods which would risk elimination of potentially useful information which was sought to be retained, given the exploratory nature of this study. Data were processed and analysed using Stata for Windows V.13.0 (College Station, Texas, USA). Kaplan-Meier curves were generated using GraphPad Prism V.6 (GraphPad Software).

## Results

After screening databases in the four involved countries, 311 patients meeting the study inclusion criteria were selected for further analysis (45% from the UK, 55% non-UK). Overall, 33% (102/311) were positive for the anti-cN-1A antibody. Table 1 shows the IBM diagnostic criteria met according to anti-cN-1A antibody status. No relationship between a diagnostic classification of ‘possible’ IBM versus ‘definite’ (for Griggs *et al* criteria) or ‘pathologically/clinically defined’ (for MRC criteria) IBM and anti-cN-1A antibody status was found (for MRC criteria, OR 0.85, 95% CI 0.48 to 1.49, p=0.565; for Griggs *et al* criteria, OR 0.70, 95% CI 0.36 to 1.36, p=0.292; analysis not performed for ENMC criteria as all anti-cN-1A antibody positive patients met the definition of ‘definite’ IBM). No difference was found in the interval between disease onset and the time of antibody testing between seropositive and seronegative groups (8.29 years (IQR 4.96–11.95) in the seropositive group vs 7.57 years (IQR 4.94–11.18) in the seronegative group, OR 1.01, 95% CI 0.97 to 1.06, p=0.604).

**Table 1:** Summary of diagnostic criteria met in patients included for analysis

Diagnostic criteria met	Anti-cN-1A positive (%)	Total (all patients)
Medical Research Council Criteria 2010^10^		
Pathologically defined IBM	13 (31.7)	41
Clinically defined IBM	39 (39.4)	99
Possible IBM	28 (33.3)	84
Griggs *et al*^9^ Criteria		
Definite IBM	19 (40.4)	47
Possible IBM	61 (32.3)	189
European Neuromuscular Centre Criteria 1997^11^		
Definite IBM	7 (31.8)	22
Probable IBM	0 (0.0)	2
Total unique patients*	102 (32.8)	311

*Some patients fulfilled multiple diagnostic criteria. Not all patients were assessed by each criterion. Of the total, 152 patients met only one criterion, 143 patients met two criteria and 16 patients met all three criteria.

Anti-cN-1A, anticytosolic 5′-nucleotidase 1A; IBM, inclusion body myositis.

### Demographics and comorbidities

No statistically significant differences were identified in demographic characteristics (including gender, age at disease onset and age at diagnosis), CK levels, smoking history or comorbidities between the anti-cN-1A antibody positive and negative groups (table 2). Non-significant trends were observed in age at disease onset and age at diagnosis (which appeared lower in the antibody-negative group) or the presence of other autoimmune diseases (which appeared more common in the antibody-positive group).

**Table 2:** Summary of demographic features, CK levels and comorbidities stratified by anti-cN-1A antibody status

	Anti-cN-1A positive	Anti-cN-1A negative	OR (95% CI)	p Value
Gender (n=311)				
Female (%)	42/102 (41.2)	84/209 (40.2)	Referent	–
Male (%)	60/102 (58.8)	125/209 (59.8)	0.96 (0.59 to 1.55)	0.868
Ethnicity (n=307)				
White (%)	97/101 (96.0)	199/206 (96.6)	Referent	–
Black (%)	2/101 (2.0)	4/206 (1.9)	1.03 (0.19 to 5.70)	0.977
Asian (%)	2/101 (2.0)	3/206 (1.5)	1.37 (0.23 to 8.32)	0.734
Other features				
Mean age in years at disease onset (SD) (n=301)	61.6 (9.7)	59.8 (9.5)	1.02 (0.99 to 1.05)	0.130
Mean age in years at diagnosis (SD) (n=305)	67.2 (9.3)	65.3 (9.5)	1.02 (1.00 to 1.05)	0.089
Disease duration in years at antibody testing (n=301)	Median 8.3 (IQR 5.0–12.0) Mean 9.0 (SD 5.5)	Median 7.6 (IQR 4.9–11.2) Mean 8.6 (SD 5.2)	1.01 (0.97 to 1.06)	0.604
Highest CK level recorded (n=223)	Median 629.0 (IQR 392–850) Mean 774.8 (SD 563.4)	Median 600.0 (IQR 400–1012) Mean 1097.2 (SD 2583.4)	1.00 (1.00 to 1.00)	0.318
Current or previous smoker (%) (n=189)	21/52 (40.4)	55/137 (40.2)	1.01 (0.53 to 1.94)	0.976
Comorbidities				
Autoimmune disease (including Sjögren's syndrome) (%) (n=244)	38/85 (44.7)	54/159 (34.0)	1.57 (0.92 to 2.70)	0.100
*Of which, Sjögren's syndrome* (%) *(n=81)*	6/33 (18.2)	8/48 (16.7)	1.11 (0.35 to 3.57)	0.859
Malignancy (%) (n=275)	12/85 (14.1)	33/190 (17.4)	0.78 (0.38 to 1.60)	0.501
Cardiovascular disease (%) (n=284)	31/91 (34.1)	64/193 (33.2)	1.04 (0.62 to 1.76)	0.880
Hypertension (%) (n=181)	29/60 (48.3)	54/121 (44.6)	1.16 (0.62 to 2.16)	0.638

‘Disease duration in years at antibody testing’ refers to the time period between disease onset and the date of anti-cN-1A antibody testing. n represents data available for analysis per variable (of a total of 311). p Value is derived from logistic regression.

Anti-cN-1A, anticytosolic 5′-nucleotidase 1A; CK, creatine kinase.

### Survival

Of the whole cohort of 311 patients, 70 deaths were recorded (31/102 (30%) in the anti-cN-1A antibody positive group and 39/209 (19%) in the negative group). The mean age of death overall was 77.8 years (SD=8.2), with no significant difference detected according to anti-cN-1A antibody status (77.0 years (SD=7.7) in the seropositive group vs 78.4 years (SD=8.6) in the seronegative group, OR 0.98, 95% CI 0.92 to 1.04, p=0.482). The cause of death was known in 63% (44 of 70) of cases. An excess of deaths as a result of respiratory disease was evident in the anti-cN-1A antibody positive group (16/25 (64%) in the anti-cN-1A antibody positive group and 9/25 (36%) in the negative group, OR 4.23, 95% CI 1.79 to 9.97, p=0.001). Adjusted analysis was not performed here due to the low numbers available for analysis. Death from other causes (cardiac, cerebrovascular, malignancy and other causes) did not differ between anti-cN-1A antibody positive and negative groups.

**Figure 1:**
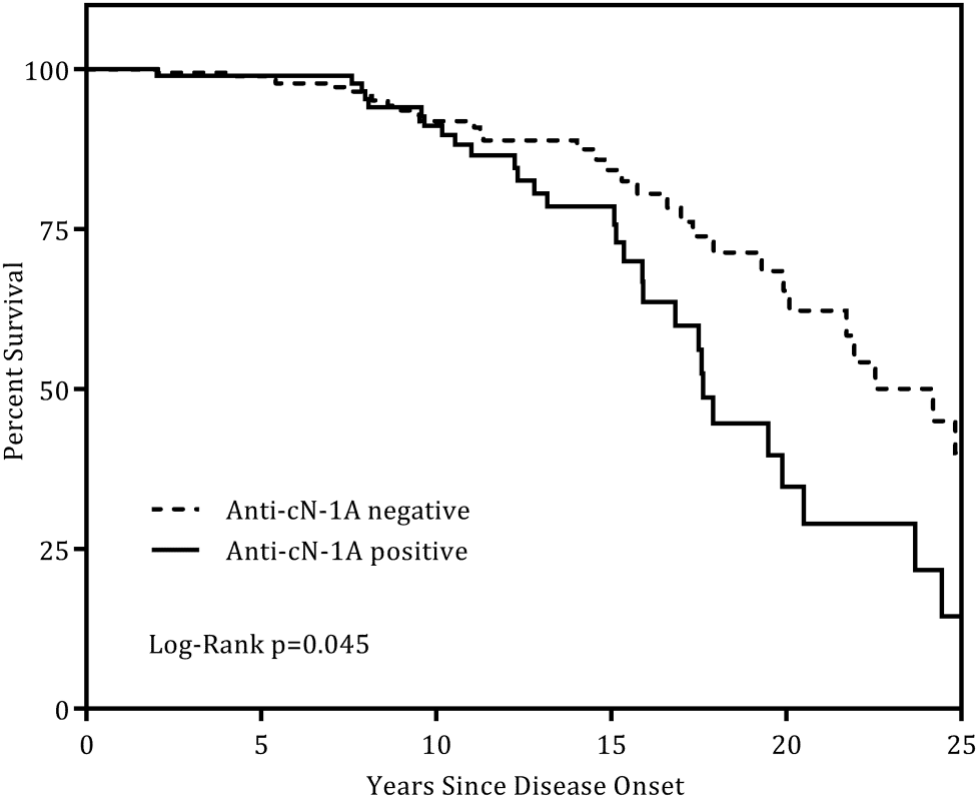
Kaplan-Meier survival curves stratified by anti-cN-1A antibody status. X-axis truncated at 25 years from disease onset.

Data from 300 patients, where the date of disease onset and date of last follow-up (or date of death) were known, were available for further analysis. This included 66 of those that had died (66/70, 94%) and comprised a total of 3550 patient-years of follow-up. The median survival in the anti-cN-1A antibody positive group was 17.6 years compared with 24.2 years in the antibody-negative group, and the Kaplan-Meier curves were significantly different (log-rank p=0.045, figure 1).

In unadjusted analysis, compared with the antibody-negative group, anti-cN-1A antibody positive patients had a 65% increased risk of death (HR 1.65, 95% CI 1.01 to 2.70, p=0.047). After adjustment for age at disease onset, gender and comorbidities, the HR was 1.95 (95% CI 1.17 to 3.27, p=0.011). Furthermore, adding the presence of dysphagia to the model confirmed an independent association (HR 1.89, 95% CI 1.11 to 3.21, p=0.019).

### Mobility

Data from 188 patients were available for this analysis. A total of 130 instances of mobility aid uptake were recorded, 81% (52/64) in the anti-cN-1A seropositive group and 63% (78/124) in the seronegative group. The overall median time between disease onset and use of a mobility aid was 8.0 years (IQR 4.6–11.0), with no significant difference between seropositive and seronegative groups (8.0 years (IQR 4.8–10.9), and 6.9 years (IQR 4.4–11.7), respectively; OR 1.01, 95% CI 0.94 to 1.08, p=0.883). Kaplan-Meier curves were not significantly different (log-rank p=0.090), so not shown. In unadjusted analysis, the HR for mobility aid requirement in the antibody-positive group was 1.35 (95% CI 0.95 to 1.93, p=0.097). After adjustment for age at disease onset, gender and comorbidities, the HR for mobility aid requirement was just outside the significance threshold (HR 1.42, 95% CI 0.99 to 2.04, p=0.056).

### Clinical features

Table 3 demonstrates the clinical characteristics at disease onset and at last clinical review, stratified by anti-cN-1A antibody status. A significant association between the presence of proximal upper limb weakness at disease onset (not a typical feature of IBM) and being anti-cN-1A antibody negative was identified (OR 0.30 95% CI 0.13 to 0.71, p=0.006). This remained significant after adjustment for age at onset, gender and comorbidities (OR 0.29, 95% CI 0.12 to 0.68, p=0.005), thus potentially defining a more classical and homogenous IBM cohort in the anti-cN-1A antibody positive group. Data regarding the presence of facial weakness were less complete (n=90). Despite this, a significantly increased incidence of facial weakness was identified in the anti-cN-1A antibody positive group at last review (OR 2.60, 95% CI 1.07 to 6.29, p=0.034), which persisted after adjustment for age at onset, gender and comorbidities (OR 3.03, 95% CI 1.20 to 7.67, p=0.019).

**Table 3:** Clinical characteristics at disease onset and at last clinical review stratified by anti-cN-1A antibody status

Clinical feature	Anti-cN-1A positive (%)	Anti-cN-1A negative (%)	OR (95% CI)	p Value
*At disease onset*				
Proximal upper limb weakness (n=252)	7/84 (8.3)	39/168 (23.2)	0.30 (0.13 to 0.71)	0.006*
Proximal lower limb weakness (n=253)	65/85 (76.5)	122/168 (72.6)	1.23 (0.67 to 2.24)	0.510
Distal upper limb weakness (n=251)	22/83 (26.5)	40/168 (23.8)	1.15 (0.63 to 2.11)	0.641
Distal lower limb weakness (n=250)	7/83 (8.4)	20/167 (12.0)	0.68 (0.27 to 1.67)	0.398
Dysphagia (n=119)	15/36 (41.7)	23/83 (27.7)	1.86 (0.82 to 4.22)	0.136
Axial involvement (n=102)	0/30 (0.0)	3/72 (4.2)	1	–
Symmetrical weakness (n=97)	25/37 (67.6)	32/60 (53.3)	1.82 (0.78 to 4.29)	0.169
*At last review*				
Proximal lower limb weakness (n=137)	35/40 (87.5)	80/97 (82.5)	1.49 (0.51 to 4.35)	0.468
Distal upper limb weakness (n=135)	40/41 (97.6)	89/94 (94.7)	2.25 (0.25 to 19.86)	0.466
Distal lower limb weakness (n=125)	23/43 (53.5)	36/82 (43.9)	1.47 (0.70 to 3.08)	0.309
Dysphagia (n=303)	63/100 (63.0)	113/203 (55.7)	1.36 (0.83 to 2.22)	0.224
Facial weakness (n=90)	18/33 (54.6)	18/57 (31.6)	2.60 (1.07 to 6.29)	0.034†
Axial involvement (n=84)	9/26 (34.6)	10/58 (17.2)	2.54 (0.88 to 7.31)	0.084
Clinical evidence of polyneuropathy (n=103)	13/38 (34.2)	31/65 (47.7)	0.57 (0.25 to 1.31)	0.184

Figures in brackets represent *within antibody group* percentages. n represents data available for analysis per variable (of a total of 311). p Value is derived from logistic regression. Data regarding certain variables (proximal upper limb weakness, facial weakness, symmetrical weakness and clinical evidence of polyneuropathy) were only available at either disease onset or at last review.

*Adjusted (for age at disease onset, gender and comorbidities) OR 0.29, 95% CI 0.12 to 0.68, p=0.005.

†Adjusted (for age at disease onset, gender and comorbidities) OR 3.03, 95% CI 1.20 to 7.67, p=0.019.

Anti-cN-1A, anticytosolic 5′-nucleotidase 1A.

### Autoantibody associations

A significant association between seropositivity for anti-SSB (La) antibodies and anti-cN-1A antibodies was identified (OR 3.28, 95% CI 1.33 to 8.07, p=0.010) (table 4). However, adjusted analysis (for anti-SSA antibodies, presence of autoimmune disorders, age at onset, gender and comorbidities) did not confirm that this association was independent (OR 2.12, 95% CI 0.52 to 8.67, p=0.297).

**Table 4:** Autoantibody profile stratified by anti-cN-1A antibody status

Antibody	Anti-cN-1A positive (%)	Anti-cN-1A negative (%)	OR (95% CI)	p Value
Antinuclear antibodies (n=132)	1/47 (2.1)	1/85 (1.2)	1.83 (0.11 to 29.88)	0.673
Anti-DNA antibodies (n=119)	3/42 (7.1)	1/77 (1.3)	5.85 (0.59 to 58.07)	0.132
Anti-Sm antibodies (n=97)	0/33 (0.0)	1/64 (1.6)	1	–
Antineutrophil cytoplasmic antibodies (n=96)	0/32 (0.0)	0/64 (0.0)	–	–
Antimitochondrial antibodies (n=128)	0/41 (0.0)	0/87 (0.0)	–	–
Antiextractable nuclear antigens antibodies (n=102)	4/34 (11.8)	5/68 (7.4)	1.68 (0.42 to 6.71)	0.463
Anti-SSA (Ro) (n=228)	19/76 (25.0)	22/152 (14.5)	1.97 (0.99 to 3.92)	0.054
Anti-SSB (La) (n=228)	13/76 (17.1)	9/152 (5.9)	3.28 (1.33 to 8.07)	0.010*
(U1)RNP antibodies (n=223)	1/74 (1.4)	0/149 (0.0)	1	–
Antitopoisomerase I (Scl70) (n=222)	0/72 (0.0)	0/150 (0.0)	–	–
Anti-Jo1 (n=228)	1/76 (1.3)	0/152 (0.0)	1	–
Other myositis-*specific* antibody (OMSA)† (n=193)	0/60 (0.0)	1/133 (0.8)	1	–
Other myositis-*associated* antibody (OMAA) (n=128)	0/41 (0.0)	0/87 (0.0)	–	–

Figures in brackets represent *within antibody group* percentages. n represents data available for analysis per variable (of a total of 311). p Value is derived from logistic regression.

*Adjusted (for anti-SSA antibodies, presence of autoimmune disorders, age at disease onset, gender and comorbidities) OR 2.11, 95% CI 0.52 to 8.67, p=0.297.

†One patient found positive for anti-SRP antibodies. In this case, no relevant clinical correlation was identified, and the relevance of this finding is uncertain.

Anti-cN-1A, anticytosolic 5′-nucleotidase 1A; OMAA, anti-Ku, anti-RNA polymerase I/II/III, anti-PM/SCL, anti-NOR90; OMSA, anti-TIF1 complex, anti-SAE, anti-NXP2, anti-MDA5, anti-SRP, anti-Mi-2, anti-PL12, anti-PL7, anti-EJ, anti-KS, anti-OJ, anti-Zo.

### Biopsy features

We identified a significant association between an excess of COX-deficient fibres on muscle biopsy and the presence of anti-cN-1A antibodies (OR 2.61, 95% CI 1.13 to 6.03, p=0.025) (table 5). In adjusted analysis (for age at disease onset, gender, comorbidities and age at biopsy), a significant independent association was confirmed (OR 2.80, 95% CI 1.17 to 6.66, p=0.020).

**Table 5:** Summary of muscle biopsy features stratified by anti-cN1-A antibody status

Biopsy feature	Anti-cN-1A positive (%)	Anti-cN-1A negative (%)	OR (95% CI)	p Value
Excess COX-deficient fibres (n=185)	53/61 (86.9)	89/124 (71.8)	2.61 (1.13 to 6.03)	0.025*
Ragged red fibres (n=164)	30/55 (54.6)	54/109 (49.5)	1.22 (0.64 to 2.34)	0.545
Atrophic fibres (n=176)	59/69 (85.5)	98/107 (91.6)	0.54 (0.21 to 1.41)	0.209
Inflammation (n=290)	94/96 (97.9)	193/194 (99.5)	0.24 (0.02 to 2.72)	0.251
MHC I upregulation (n=198)	67/69 (97.1)	124/129 (96.1)	1.35 (0.26 to 7.15)	0.724
Necrosis (n=136)	40/50 (80.0)	61/86 (70.9)	1.64 (0.71 to 3.78)	0.246
Mononuclear infiltrate (n=224)	72/74 (97.3)	143/150 (95.3)	1.76 (0.36 to 8.70)	0.487
Invasion of non-necrotic fibres (‘partial invasion’) (n=95)	21/30 (70.0)	48/65 (73.9)	0.83 (0.32 to 2.15)	0.696
Rimmed vacuoles (n=257)	77/88 (87.5)	143/169 (84.6)	1.27 (0.60 to 2.72)	0.533
Protein deposits† (n=128)	24/44 (54.6)	53/84 (63.1)	0.70 (0.34 to 1.47)	0.349
Microfilaments‡ (n=81)	9/24 (37.5)	24/57 (42.1)	0.83 (0.31 to 2.20)	0.700

Figures in brackets represent *within antibody group* percentages. n represents data available for analysis per variable (of a total of 311). p Value is derived from logistic regression.

*Adjusted (for age at disease onset, gender and comorbidities) OR 2.60, 95% CI 1.11 to 6.12, p=0.028. Adjusted (additionally for age at biopsy) OR 2.80, 95% CI 1.17 to 6.66, p=0.020.

†Includes amyloid (Congo Red or immunofluorescence), p62 (immunofluorescence) and TDP-43 (immunofluorescence).

‡15–21 nm tubulofilaments identified by electron microscopy.

Anti-cN-1A, anticytosolic 5′-nucleotidase 1A; COX, cytochrome oxidase; MHC, major histocompatibility complex.

## Discussion

This multinational exploratory study represents the first of its kind to combine analysis of clinical, histopathological, other serological and mortality data in a large cohort of patients with IBM stratified according to anti-cN-1A antibody status. Our results will guide future confirmatory studies and highlight potential disease mechanisms warranting further evaluation. We found that the anti-cN-1A antibody positive group had a significantly increased mortality risk independent of age, gender, comorbidities and the presence of dysphagia. We also found a smaller proportion with proximal upper limb weakness at disease onset and an excess of COX-deficient fibres on muscle biopsy in the anti-cN-1A antibody positive group. An increased likelihood of having facial weakness and an association between antibody positivity and death from a respiratory cause was also observed, although the numbers assessed here were small. As in other studies, we did not find a relationship between disease duration and the likelihood of identifying anti-cN-1A antibodies.^18^ ^19^

There are limited reports in the literature comparing the characteristics of patients with IBM with and without anti-cN-1A antibodies, amounting to 258 patients in four separate studies.^18^ ^19^ ^24^ ^25^ A small proportion of the cases analysed here was included in a previous analysis which did not focus on differences on clinical characteristics according to serotype.^22^ Some authors identified no significant differences in the characteristics between cohorts, whereas others have suggested that the anti-cN-1A antibody positive group exhibits a more severe phenotype.^18^ ^19^ Lloyd *et al*^24^ identified a lower incidence of rimmed vacuoles on biopsy in those without anti-cN-1A reactivity but with no clinical differences between the studied cohorts, findings that were not replicated here. A very recent study found no differences between 24 cN-1A seropositive and 45 seronegative patients with IBM regarding class II human leukocyte antigen (HLA) alleles and the presence of other antibodies.^25^

The simultaneous discovery of anti-cN-1A antibodies in 2011 by two independent research groups offers potential insights into the pathogenesis of IBM, and will contribute to the debate about the relative influence of the immune system and degeneration.^16^ ^19^ ^23^ The presence of anti-cN-1A in other autoimmune diseases such as Sjögren's syndrome is also of interest as it might highlight shared underlying immune mechanisms across these diseases.^22^ As with most other MSAs, further research is required to establish the mechanisms involved in anti-cN-1A reactivity in IBM.

Anti-cN-1A antibodies are present in the sera of 29%–52% of patients with IBM (33% in our cohort).^16^ ^17^ Higher proportions of anti-cN-1A antibody seropositivity in other studies (up to 72%) might be explained by different techniques used in different centres, by different cut-off levels for positivity or by differences in patient selection.^18^ The current study used very strict cut-off values in ELISA testing.^26^ In a recent study, anti-cN-1A antibodies were found in 37% of patients with IBM, compared with <5% in PM, DM and other neuromuscular disorders, highlighting a potential utility of using anti-cN-1A antibody testing to differentiate IBM and mimicking diagnoses.^22^ However, the specificity of testing is limited by a high reactivity in some other autoimmune and connective tissue diseases (in 36% of patients with Sjögren's syndrome and in 20% with systemic lupus erythematosus).^22^ ^24^

The higher frequency of COX-negative fibres, a feature of mitochondrial dysfunction, indicates possible differences in molecular pathways within the subgroups defined by anti-cN-1A antibody status. The reasons for increased mortality and the suggestion of increased risk of death from respiratory cause are unexplained, but these findings appear to agree with those of Goyal *et al*^18^ who also found a more severe respiratory phenotype in the antibody-positive group. The lower frequency of proximal upper limb weakness at presentation in the anti-cN-1A antibody positive compared with antibody-negative group remains unexplained.

This study represents the largest cohort of patients with IBM and has only been achieved by an international collaborative effort. Established IBM diagnostic criteria were used to include patients for the analysis, a predefined set of clinical data was retrieved in each patient and all anti-cN-1A testing was performed in one laboratory. However, there remain a number of limitations. The study was retrospective and relied on the identification and recording of clinical characteristics by the treating physicians. In the UK cohort, the recruiting physician (the patient's treating consultant neurologist or rheumatologist) was asked to recall the symptoms that were present at the time of disease onset when completing the pro forma at the time of recruitment, and as such these details may be subject to recall bias. While efforts to minimise missing data were made, data were not complete for all study parameters in all cases, although there was no evidence to suggest that this occurred in a systematic way. Analysis involved pooling of data from different cohorts. There is potential for differences in data collection methodology between cohorts (see online supplementary appendix 1) to reduce the reliability of our findings. However, a comparison of features between UK and non-UK cohorts where pooled data were analysed has revealed largely comparable findings (see online supplementary table S1). Overall, we feel that our pooled analysis has increased statistical power and reduced the likelihood of statistical errors occurring. Objective measurements of muscle strength (eg, dynamometry of the finger flexors) could have improved sensitivity of detection of weakness, but such methods were not available. Also, this study did not perform a reanalysis of muscle biopsy tissue. The cause of death was difficult to establish in some patients in the non-UK cohort, due to missing information in the medical records, and in the UK cohort due to an inability to match some patients to the nationally stored mortality data held by the UK Health and Social Care Information Centre.

10.1136/annrheumdis-2016-210282.supp2 supplementary table

In the future, anti-cN-1A autoantibody testing and anti-cN-1A autoantibody status could be used in the diagnostic workup of potential IBM cases, and there remains the opportunity to use anti-cN-1A antibody status in the construction of future diagnostic criteria for IBM. However, the results of the current study also suggest that distinct IBM subtypes may be identified according to anti-cN1-A antibody status. Therefore, serum anti-cN-1A testing might also be of use in the stratification of patients with IBM (eg, for clinical trials), rather than purely as a diagnostic biomarker. A large prospective study with a sufficient duration of follow-up might offer potential to further investigate the overall utility of anti-cN-1A antibody testing in the clinical and research settings.

## Conclusion

In this exploratory study, comparison of patients with IBM with and without anti-cN-1A autoantibody reactivity identified differences in their mortality risk, clinical characteristics and histopathological findings. The largest study of its kind has demonstrated that anti-cN-1A antibody testing may, and over and above its diagnostic value, be clinically useful to define distinct IBM subtypes.
