# Supplementary material for: Cytosolic 5′-nucleotidase 1A autoantibody profile and clinical characteristics in inclusion body myositis
Source: Ann Rheum Dis. 2017 Jan 25;76(5):862–8. doi: 10.1136/annrheumdis-2016-210282 (PMC5530338; doi:10.1136/annrheumdis-2016-210282)
Supplement: supplementary appendix [file annrheumdis-2016-210282supp001.pdf]

Supplementary Appendix 1 - Standardized data collection pro-forma

French data collection pro-forma: computerized: MACS (translated from French)

Theme	Items	Items choice
Identification/ patient characteristics	Treating physician	[open field]
	Investigator's name	[open field]
	Birthplace	[open field]
	Profession	[open field]
	Ethnicity	North Africa, Asia, Caucasian, Sub-Saharan Africa, Other (non specified)
	Name	[open field]
	Date of Birth	Date
	Gender	Male/Female
Database	Date of data extraction	Date
	Diagnosis	Choose from:<br>PM,<br>anti-synthetase,<br>PM without antibodies or extramuscular symptoms,<br>DM,<br>auto-immune necrotizing myopathy,<br>IBM,<br>Toxic myopathy, granulomatous myositis, fasciitis,<br>cortisone induced myopathy,<br>distal myositis,<br>focal myositis,<br>other (non specified), unknown
	Diagnostic criteria	Presence/absence/unknown indicated for each of the following items:<br>- elevated CK<br>- muscle weakness<br>- EMG with myopathic changes<br>- myositis or necrotizing myopathy on biopsy<br>- characteristic skin lesions
	IBM	Choose from:<br>Certain diagnosis of IBM, overlap IBM/PM,<br>PM with COX negative fibers
Symptoms	Were muscular symptoms ever present?	Yes/no/unknown

	Extra muscular symptoms	Yes/No/unknown
	Most altered lung function test	Yes/No/unknown: if yes: date and results
	Cardiac symptoms	Yes/No/unknown: if yes: specify
	Rheumatic complaints	Arthralgia Yes/No/unknown;<br>and:<br>Arthritis Yes/No/unknown
	Raynaud phenomenon	Yes/No/unknown
	Digestive tract problems	Yes/No/unknown: if yes: specify
	Thrombosis	Yes/No/unknown; if yes: arterial or venous; recurrence yes/no
	Renal symptoms	Yes/No/unknown: if yes: specify
	Cancer in 3 years preceding diagnosis of myositis	Yes/No/unknown: if yes: specify the tissue involved and date of diagnosis
	Infections	HIV yes/no, hepatitis B yes/no, hepatitis C yes/no
	Neurological symptoms	Yes/No/unknown; if yes: specify
	Exposition to toxins	Amiante (asbestos), silice (silica), glass fibre, solvents, carbon yes/no
	Use of statins	Yes/No/unknown; if yes: specify duration of treatment and name of medicine
	Other expositions	Fibrates, hydroxy-urea, chloroquine, D-penicillamine yes/no
	Tobacco use	Yes/No/unknown: if yes: still smoking, smoking in the past, quit smoking>1 year ago, unknown; + estimation of nr of package years
	Sports	Regular sport in past Yes/No/unknown
	Pulmonary CT performed	Yes/No/unknown: if yes: date and result
	Pulmonary symptoms	Yes/No/unknown: if yes:<br>- interstitial pneumopathy yes/no<br>- diaphragm yes/no<br>- pulmonary hypertension yes/no<br>- inhalation pneumopathy yes/no
Treatment	Treatment used	Dosage, start and end date inserted
	Treatment ongoing	Yes/no
Family History	Connective tissue disorder	Yes/no/unknown
	Dystrophy	Yes/no/unknown

Onset	Date of onset of symptoms	date
	Duration of symptoms	In years
	Date of first visit in outpatient clinic	date
	Duration of follow up	In years
	Date of diagnosis	Date
	Age at moment of diagnosis	In years
Muscle biopsy	Normal	Yes/No/unknown
	Mitochondrial abnormalities	Yes/No/unknown
	Abnormalities electron microscopy	Yes/No/unknown
	Abnormalities of vessels	Yes/No/unknown
	Atrophic fibers	Yes/No/unknown
	Date of biopsy	date
	Inflammation	Yes/no/unknown
	Does pathologists conclusion correspond with diagnosis of myositis	Yes/No/unknown
Laboratory tests	CK elevated	Yes/No/unknown
	Highest level of CK	
	MSA present	Yes/No/unknown for each of the following:<br>Jo1<br>PL7<br>PL12<br>OJ<br>EJ<br>Mi2<br>MDA5<br>TIF1gamma<br>HMGCoAR<br>SRP<br>SAE1<br>SAE2<br>NXP2
	MAA present	Yes/No/unknown for each of the following:<br>ANA<br>SSA/Ro52<br>SSA/Ro60<br>SSB<br>RNP

		Sm<br>Anti-Scl70<br>anti-centromere<br>PM-Scl<br>Ku<br>Anti-DNA<br>ANCA<br>Anti-mitochondrial<br>Rheumatoid factor<br>CCP
	Other autoantibody	Yes/No/unknown<br>(if yes: specify)
Other diagnostic tests	Muscle MRI performed	Yes/No/unknown: if yes: date and results: fatty infiltration T1 yes/no/unknown; hyperintense signal T2 yes/no/unknown; contrast enhancement yes/no/unknown

Dutch pro-forma used

Applied to Swedish and Dutch population; in French population both French pro-forma and Dutch pro-forma were used in each patient.

Theme	Items	Items choice
Identification/ patient characteristics	Casenumber	[open field]
	Ethnicity	Africa, Asia, Caucasian, Hispanic, Mix, unknown
	Date of Birth	Date
	Gender	Male/Female
Database	Date of data collection	Date
Symptoms/ Prognosis	Status	Alive/death/unknown
	Date of last follow up of alive patients; date of death of dead patients	Date
	Age of death	In years
	Duration between onset of symptoms and death	In years
	Cause of death	Choose from following options:<br>- pulmonary (infection, respiratory failure, other – non specified)<br>- cardiac (infarction, rhythm abnormalities, other – non specified)<br>- stroke or other cerebrovascular disease<br>- other known cause<br>- unknown cause
	First symptoms	Choose from:<br>- proximal weakness<br>- distal weakness<br>- dysphagia<br>- facial weakness<br>- other (non specified)
	Symmetry at onset	Symmetric/asymmetric/unknown
	Proximal arm weakness at onset	Yes/No/unknown
	Proximal leg weakness at onset	Yes/No/unknown
	Distal arm weakness at onset	Yes/No/unknown
	Proximal arm weakness at onset	Yes/No/unknown

	Dropped foot at onset	Yes/No/unknown
	Atrophy at onset	Yes/No/unknown
	Facial weakness at onset	Yes/No/unknown
	Myalgia at onset	Yes/No/unknown
	Deep tendon reflexes upper extremity	Normal/diminished or absent/unknown
	Deep tendon reflexes lower extremity	Normal/diminished or absent/unknown
	Fulfilling Griggs criteria?	Definite IBM / Possible IBM / no IBM / unknown
	Fulfilling Hilton-Jones criteria?	Pathologically defined IBM / clinically defined IBM / Possible IBM / No IBM / unknown
Onset	Date of onset of symptoms	date
	Duration of symptoms	In years
	Date of first visit in outpatient clinic	date
	Duration of follow up	In years
	Date of diagnosis	Date
	Age at moment of onset of symptoms	In years
Last follow up	Time between IBM diagnosis and last follow up	In years
	Age at last follow up	In years
	Pattern of weakness at last follow up	Choose between:<br>- only proximal weakness, symmetrical<br>- only proximal weakness, asymmetrical<br>- only distal weakness, symmetrical<br>- only distal weakness, asymmetrical<br>- both proximal and distal weakness, symmetrical<br>- both proximal and distal weakness, asymmetrical<br>- no weakness<br>- unknown
	Polyneuropathy at last follow-up	Present based on clinical features / present based on electromyography and clinical features / absent / unknown
	Dysphagia at last follow	Yes/no/unknown

	up	
	Facial weakness at last follow up	Yes/no/unknown
	Dropped foot at last follow up	Yes/no/unknown
	Dropped head at last follow up	Yes/no/unknown
	Camptocormia at last follow up	Yes/no/unknown
	Ankylosis/contracture at last follow up	Yes/no/unknown
	Deep tendon reflexes upper extremity at last follow up	Normal/diminished or absent/unknown
	Deep tendon reflexes lower extremity at last follow up	Normal/diminished or absent/unknown
Mobility	Number of falls	None / up to twice a year / up to once a month / unknown
	Mobility aids	Use of wheelchair at last follow up / use of walker or cane at last follow up / no mobility aids / unknown
	Time between onset of symptoms and start using wheelchair	In months
	Time between onset of symptoms and start using cane or walker	In months
Muscle biopsy	Mononuclear infiltrate	Yes/No/unknown
	COX negative fibers exceeding local normal values according to age	Yes/No/unknown
	Microfilaments	Yes/No/unknown
	Protein deposits	Yes/No/unknown
	Atrophic fibers	Yes/No/unknown
	MHC-I or HLA-B positivity	Yes/no/unknown
	Rimmed vacuoles	Yes/No/unknown
	Inflammation	Yes/No/unknown
	Necrotic fibers	Yes/No/unknown
	Date of biopsy	Date
Laboratory tests	CK level at diagnosis	
	Highest level of CK	
	MSA present	Yes/No/unknown
	MAA present	Yes/No/unknown for each of the following:

		ANA<br>SSA<br>SSB<br>RNP<br>Sm<br>Anti-Scl70<br>Anti-dsDNA<br>ANCA
Other diagnostic tests	EMG performed	Normal / myopathic / neurogenic / mixed pattern / not performed / unknown
	Other lab tests	Level of total cholesterol, HDL cholesterol, triglycerides. Presence or absence of paraproteins in blood, CSF abnormalities, brain MRI, apolipoprotein e-alleles abnormalities
	Muscle MRI performed	Yes/No/unknown: if yes: date and results: fatty infiltration T1 yes/no/unknown; hyperintense signal T2 yes/no/unknown; contrast enhancement yes/no/unknown
Treatment	Which treatment used?	Choose between:<br>- none/supportive treatment<br>- corticosteroids<br>- iv immunoglobulins<br>- azathioprine<br>- methotrexate<br>- cyclophosphamide<br>- anakinra<br>- BYM338<br>- combination of upper treatments<br>- unknown
	Duration of treatment	In months
	Effect of treatment	None / reduction of symptoms / slowing of progression / unknown
Comorbidity	Other autoimmune disease	Present/absent/unknown
	Cardiovascular disease (including cardiomyopathy)	Present/absent/unknown
	Hypertension (<140/90mmHg at >3 different time points)	Present/absent/unknown

UK pro-forma

In this subgroup, the following data fields were extracted from the UKMYONET registry (data stored using EuroMyositis platform - https://euromyositis.eu/)

Field name	Options		
	Core dataset		
Sex	Male	Female	
Ethnic origin (Select mixed race by selecting more than one)	Caucasian	Afro-Caribbean	Black African
	Asian	Oriental	Hispanic
	Ethnic origin (Other)		
Date of birth			
Month and year of first symptoms of myositis			
Month and year of diagnosis			
Malignancy (regardless of whether considered to be myositis associated) (Site/Tissue type. Select one or more or specify in other field below)	Bladder	Bowel	Breast
	Gastric	Hepatic	Kidney
	Lung	Lymphoma	Metastatic
	Other (specify)	Ovarian	Pancreas
	Prostate	Skin	Tongue
	Uterine	Vulva	
Month/year of cancer diagnosis			
Myositis-associated interstitial lung disease	Yes	No	N/A
Cardiac involvement due to myositis	Yes	No	N/A
Dysphagia	Yes	No	N/A
More severe weakness of forearm flexors than of forearm extensors	Yes	No	N/A
Finger flexor weakness	Yes	No	N/A
Quadriceps muscle weakness (≤ grade 4 MRC)	Yes	No	N/A
Ever smoked	Yes	No	N/A
	IBM dataset		
Diagnostic criteria	Griggs Definite	Griggs Possible	
	Verschuuren Definite	Verschuuren Probable	Verschuuren Possible
	ENMC Definite	ENMC Probable	
	MRC Pathologically defined	MRC Clinically defined	MRC Possible
Creatine Kinase	NR	AB	UK
	Highest value:		
**Initial symptoms and signs**			

a) Proximal upper extremity involvement	Yes	No	N/A
b) Distal upper extremity involvement	Yes	No	N/A
c) Proximal lower extremity involvement	Yes	No	N/A
d) Distal lower extremity involvement	Yes	No	N/A
e) Initial Dysphagia	Yes	No	N/A
f) Axial involvement	Yes	No	N/A
g) Respiratory muscle weakness	Yes	No	N/A
**Current Clinical Features**			
a) Slowly progressive course	Yes	No	N/A
b) Sporadic disease	Yes	No	N/A
c) Muscle weakness affecting proximal and distal muscles of arms & legs	Yes	No	N/A
d) Finger flexor weakness	Yes	No	N/A
e) Quadriceps muscle weakness (< 4 MRC)	Yes	No	N/A
f) Weakness of knee extension > hip flexion	Yes	No	N/A
g) Weakness of finger flexion > shoulder abduction	Yes	No	N/A
h) Wrist flexor > wrist extensor weakness	Yes	No	N/A
I) Lack of stabilization / remission with immunosuppression after one year of treatment	Yes	No	N/A
j) Presence of other autoimmune diseases	Yes	No	N/A
If so specify:			
Family history of neuromuscular disease	Yes	No	N/A
If so specify:			
**Biopsy features**			
Endomysial inflammatory infiltrates	Yes	No	N/A
MHC-I expression on sarcolemma	Yes	No	N/A

Inflammatory cell infiltration characterized by mononuclear cell invasion of non-necrotic muscle fibres	Yes	No	N/A
Rimmed vacuoles in non-necrotic fibres	Yes	No	N/A
Intracellular amyloid deposits	Yes	No	N/A
Hyperphosphorylated tau (SMI-31) immunoreactivity	Yes	No	N/A
p62 immunoreactivity	Yes	No	N/A
TDP-43 immunoreactivity	Yes	No	N/A
15-21nm tubulofilaments by electron microscopy	Yes	No	N/A
Cytochrome oxidase deficient fibres (abnormal for age)	Yes	No	N/A
Ragged red fibres	Yes	No	N/A

In addition, the following data was collected from the patients case notes where available:

Field name	Options				
Walking aid requirement status	Y	N	Unknown	Not Required	Date
Wheelchair use status	Y	N	Unknown	Not Required	Date
Comorbid cardiovascular disease	Y	N	Unknown		
Comorbid hypertension	Y	N	Unknown		

Further data regarding mortality (Dead/Alive, date status confirmed) and the occurrence of malignancy (Y/N, Type/Site, date diagnosed) for the UK cohort were obtained from the UK Health and Social Care Information Centre (now NHS Digital - https://digital.nhs.uk/).
