# Supplementary material for: Cytosolic 5′-nucleotidase 1A autoantibody profile and clinical characteristics in inclusion body myositis
Source: Ann Rheum Dis. 2017 Jan 25;76(5):862–8. doi: 10.1136/annrheumdis-2016-210282 (PMC5530338; doi:10.1136/annrheumdis-2016-210282)
Supplement: supplementary table [file annrheumdis-2016-210282supp002.pdf]

Supplementary Table 1

A comparison of features between the UK and non-UK cohort where anti-cN-1A antibodies were detected. Variables are included in this table where data was available from both cohorts which were pooled for combined analysis. Two-sided p-values have been generated from Fisher's exact test, Students t-test or the Wilcoxon rank sum test where appropriate. "n" refers to the number of anti-cN-1A antibody patients as a proportion of all patients.

	**Anti-cN-1A Positive (UK)**	**Anti-cN-1A Positive (non-UK)**	**p**
**Totals (%) (n=102/311)**	41/141 (29.1)	61/170 (35.9)	0.226
Gender (n=102/311)			
Female (%)	15/41 (36.6)	27/61 (44.3)	0.539
Male (%)	26/41 (63.4)	34/61 (55.7)	0.539
Ethnicity (n=101/307)			
White (%)	39/41 (95.1)	58/60 (96.7)	0.143
Black (%)	0/41 (0)	2/60 (3.3)	0.143
Asian (%)	2/41 (4.9)	0/60 (0)	0.143
Other features			
Mean age in years at disease onset (SD) (n=99/301)	62.0 (11.5)	61.3 (8.3)	0.754
Mean age in years at diagnosis (SD) (n=100/305)	66.7 (10.9)	67.6 (8.1)	0.609
Median disease duration in years at antibody testing (IQR) (n=99/301)	8.3 (4.7-10.4)	8.4 (5.8-13.0)	0.171
Median highest CK level recorded (IQR) (n=77/223)	737 (417-813)	596.5 (370-1010)	0.701
Current or previous smoker (%) (n=52/189)	11/34 (32.4)	10/18 (55.6)	0.141
**Comorbidities**			
Autoimmune disease (including Sjögren's syndrome) (%) (n=85/244)	14/30 (46.7)	24/55 (43.6)	0.823
Of which, Sjögren's syndrome (%) (n=33/81)	5/14 (35.7)	1/19 (5.3)	0.062
Malignancy (%) (n=85/275)	7/40 (17.5)	5/45 (11.1)	0.535
Cardiovascular disease (%) (n=91/284)	5/34 (14.7)	26/57 (45.6)	0.003
Hypertension (%) (n=60/181)	9/17 (52.9)	20/43 (46.5)	0.777
Clinical Features at Disease Onset			
Proximal Upper Limb Weakness (%) (n=84/252)	1/32 (3.1)	6/52 (11.5)	0.243
Proximal Lower Limb Weakness (%) (n=85/253)	22/33 (66.7)	43/52 (82.7)	0.117
Distal Upper Limb Weakness (%) (n=83/251)	14/32 (43.8)	8/51 (15.7)	0.010
Distal Lower Limb Weakness (%) (n=83/250)	5/32 (15.6)	2/51 (3.9)	0.102
Dysphagia (%) (n=36/119)	11/32 (34.4)	4/4 (100)	0.023‡
Clinical Features at Last Review			
Dysphagia (n=100/303)	23/41 (56.1)	40/59 (67.8)	0.293
Antibody status			
Anti-SSA (Ro) (%) (n=76/228)	9/41 (22.0)	10/35 (28.6)	0.599
Anti-SSB (La) (%) (n=76/228)	5/41 (12.2)	8/35 (22.9)	0.240
(U1)RNP antibodies (%) (n=74/223)	0/41 (0)	1/33 (3.0)	0.446
Anti-Topoisomerase I (Scl70) (%) (n=72/222)	0/41 (0)	0/31 (0)	-
Anti-Jo1 (%) (n=76/228)	0/41 (0)	1/35 (2.9)	0.461
Other Myositis *Specific* Antibody (OMSA) (%) (n=60/193)	0/41 (0)	0/19 (0)	-
Biopsy Feature			
Excess COX deficient fibers (n=61/185)	24/26 (92.3)	29/35 (82.9)	0.448
Ragged red fibers (n=55/164)	6/18 (33.3)	24/37 (64.9)	0.043
Atrophic fibers (n=69/176)	8/13 (61.5)	51/56 (91.1)	0.016
Inflammation (n=96/290)	37/39 (94.9)	57/57 (100)	0.162
MHC I upregulation (n=69/198)	26/26 (100)	41/43 (95.4)	0.523
Mononuclear infiltrate (n=74/224)	31/31 (100)	41/43 (95.4)	0.506
Rimmed vacuoles (n=88/257)	24/31 (77.4)	53/57 (93.0)	0.047
Protein deposits* (n=44/128)	16/23 (69.6)	8/21 (38.1)	0.068
Microfilaments** (n=24/81)	3/6 (50.0)	6/18 (33.3)	0.635

‡in the non-UK cohort, data regarding dysphagia at disease onset was only available in a small subgroup.
